# Salidroside may target PPARα to exert preventive and therapeutic activities on NASH

**DOI:** 10.3389/fphar.2024.1433076

**Published:** 2024-10-02

**Authors:** Xueru Chu, Shousheng Liu, Baozhen Qu, Yongning Xin, Linlin Lu

**Affiliations:** ^1^ Department of Infectious Disease, Qingdao Municipal Hospital, School of Medicine and Pharmacy, Ocean University of China, Qingdao, China; ^2^ Department of Infectious Disease, Qingdao Municipal Hospital, Qingdao, China; ^3^ Clinical Research Center, Qingdao Municipal Hospital, University of Health and Rehabilitation Sciences, Qingdao, China; ^4^ Qingdao Cancer Prevention and Treatment Research Institute, Qingdao Central Hospital, University of Health and Rehabilitation Sciences, Qingdao, China

**Keywords:** salidroside, non-alcoholic steatohepatitis, prevention, therapy, immunity, inflammation, PPARα

## Abstract

**Background:**

Salidroside (SDS), a phenylpropanoid glycoside, is an antioxidant component isolated from the traditional Chinese medicine *Rhodiola rosea* and has multifunctional bioactivities, particularly possessing potent hepatoprotective function. Non-alcoholic steatohepatitis (NASH) is one of the most prevalent chronic liver diseases worldwide, but it still lacks efficient drugs. This study aimed to assess the preventive and therapeutic effects of SDS on NASH and its underlying mechanisms in a mouse model subjected to a methionine- and choline-deficient (MCD) diet.

**Methods:**

C57BL/6J mice were fed an MCD diet to induce NASH. During or after the formation of the MCD-induced NASH model, SDS (24 mg/kg/day) was supplied as a form of diet for 4 weeks. The histopathological changes were evaluated by H&E staining. Oil Red O staining and Sirius Red staining were used to quantitatively determine the lipid accumulation and collagen fibers in the liver. Serum lipid and liver enzyme levels were measured. The morphology of autophagic vesicles and autophagosomes was observed by transmission electron microscopy (TEM), and qRT-PCR and Western blotting were used to detect autophagy-related factor levels. Immunohistochemistry and TUNEL staining were used to evaluate the apoptosis of liver tissues. Flow cytometry was used to detect the composition of immune cells. ELISA was used to evaluate the expression of serum inflammatory factors. Transcript–proteome sequencing, molecular docking, qRT-PCR, and Western blotting were performed to explore the mechanism and target of SDS in NASH.

**Results:**

The oral administration of SDS demonstrated comprehensive efficacy in NASH. SDS showed both promising preventive and therapeutic effects on NASH *in vivo*. SDS could upregulate autophagy, downregulate apoptosis, rebalance immunity, and alleviate inflammation to exert anti-NASH properties. Finally, the results of transcript–proteome sequencing, molecular docking evaluation, and experimental validation showed that SDS might exert its multiple effects through targeting PPARα.

**Conclusion:**

Our findings revealed that SDS could regulate liver autophagy and apoptosis, regulating both innate immunity and adaptive immunity and alleviating inflammation in NASH prevention and therapy via the PPAR pathway, suggesting that SDS could be a potential anti-NASH drug in the future.

## 1 Introduction

Non-alcoholic fatty liver disease (NAFLD) is the most common chronic liver disease with the prevalence increasing yearly, affecting the health of both adults and children (Younossi et al., 2018). NAFLD is a clinicopathologic syndrome characterized by excessive fat deposition in hepatocytes caused by alcohol and other specific liver damaging factors and an acquired metabolic stress liver injury closely related to insulin resistance and genetic susceptibility. NAFLD consists of a series of hepatic abnormalities extending from a non-alcoholic fatty liver (NAFL) to non-alcoholic steatohepatitis (NASH) with possible developments of liver fibrosis, cirrhosis, and liver cancer (Qu et al., 2022). NASH is considered a hallmark of the progression and deterioration of NAFLD and is characterized by hepatic steatosis, liver cell damage, innate immune cell-mediated inflammation, and varying degrees of fibrosis. The “two-hit” mechanism is the most widely accepted theory to explain the development of NAFLD to NASH. The first hit is mainly caused by the excessive accumulation of lipids in the liver, which is closely associated with mitochondrial disorders caused by lipotoxicity, making the liver sensitive to the second hit. The second hit is related to a combination of inflammatory response, oxidative stress, liver damage, and fibrosis (Gu et al., 2023). Without intervention, the continuous mechanisms of tissue damage and regeneration typical of NASH chronic inflammation can progress to liver fibrosis, cirrhosis, or even hepatocellular carcinoma (Motta et al., 2023). The prevalence of NAFLD among the general population is approximately 20%–25%, whereas the prevalence of NASH is expected to increase by up to 56% by 2030 in China, Europe, Japan, the United Kingdom, and the United States (Motta et al., 2023; Llovet et al., 2023). Recent studies indicated that 20% of the patients with NAFLD histologically show NASH in biopsy specimens (Han S. K. et al., 2023). Due to the increasing prevalence of NASH, it is considered the second most common cause of liver transplantation in the United States after chronic hepatitis C (Aldossari, 2023). The annual incidence of hepatocellular carcinoma (HCC) in patients with NASH-related cirrhosis is about 2%, and about 35%–50% of HCC in NASH occurs in patients with cirrhosis and before routine cancer screening (Llovet et al., 2023; Liu and Chien, 2023). Overall, NASH is associated with an increased risk of HCC and mortality and is expected to become the leading cause of HCC worldwide by 2030 (Quek et al., 2023). However, there are currently no approved preventive treatments or treatments for NASH beyond lifestyle changes (Harrison et al., 2023). NASH is strongly associated with metabolic disorders and obesity. At present, dietary intervention is considered one of the main strategies to prevent NASH, and the development of safe and effective NASH drugs has long-term social and economic significance.

It is well-known that traditional herbal medicines have been used for centuries to cure people with many diseases, and they are essential sources for originating hepatoprotective drugs. Several therapeutic candidate drugs, such as silymarin and berberine, have been selected and verified from these traditional herbs, and they have been in the fourth phase of clinical research for the treatment of NAFLD (Yan et al., 2020). *Rhodiola rosea* (*hong jing tian* in Chinese) is a prestigious plant used in Chinese traditional medicine application for its hepatoprotective and neuroprotective function. Salidroside (SDS), a phenylpropanoid glycoside, is an antioxidant component isolated from *R. rosea* (Fan et al., 2020). Numerous studies have shown that SDS possesses anti-hypoxia, anti-fatigue, anti-viral, anti-cancer, anti-inflammatory, immune-balancing, and lipid-lowering effects, glucose and lipid metabolism improvement, and many other properties both *in vitro* and *in vivo* (Gao et al., 2023; Hu et al., 2021; Rong et al., 2020). In terms of hepatoprotective function, SDS has a protective effect on various types of liver injury, such as alleviating chemical liver injury through the Sirt1-mediated Akt/Nrf2 pathway (Xu et al., 2023), improving inflammation in alcohol-induced liver injury through TLR4/TAK1 (Sun et al., 2016), regulating GSK-3β/Nrf2 to protect against liver ischemia–reperfusion injury (Cai et al., 2017), facilitating the MIF pathway and downstream hippophagia and lipid metabolism to remission NAFLD (Liu et al., 2022), activating the AMP to suppress NASH in mice (Hu et al., 2021), preventing immune-mediated hepatitis in mice (Hu et al., 2014), alleviating liver fibrosis through the NF-κB and TGF-β1/Smad3 pathway (Feng et al., 2018), and inhibiting the activation of the Notch1 signaling pathway to inhibit liver malignant tumor (Lu et al., 2019). However, few studies have investigated the efficacy of the dietary intake of SDS on the prevention and therapy of NASH, and the immune-boosting effects of SDS on NASH are poorly understood. As mentioned above, the “two-hit” mechanism leads to liver lipid accumulation, lipid toxicity, inflammatory response, cell apoptosis, and fibrosis, which are the main reasons for the progression of NASH. In addition, the innate and adaptive immune responses involved in NASH are other important factors promoting liver inflammation and cell injury (Tilg and Moschen, 2010). Hence, exploring an ideal drug that can regulate multiple pathogenetic pathways of NASH will contribute to achieving an effective therapy response to NASH.

Peroxisome proliferator-activated receptors (PPARs) are ligand-activated transcription factors belonging to the nuclear hormone receptor superfamily, which are proven effective therapeutic targets for NASH treatment. There are three identified isoforms of PPARs (α, β/δ, and γ), all of which are involved in lipid metabolism and glucose homeostasis in NAFLD/NASH (Montagner et al., 2016). In addition, PPARs exist in different types of immune cells and may modulate both hepatic and systemic inflammatory responses (Staels et al., 2023). Thus, PPARs can simultaneously regulate different interrelated mechanisms of NASH pathogenesis due to their regulating whole-body lipid and glucose metabolism and inflammation functions.

In this study, we first investigated and evaluated the potential preventive and protective effects of the dietary administration of SDS in a mouse model of NASH induced by a methionine- and choline-deficient (MCD) diet and then explored the molecular mechanism and target of SDS by transcriptome–proteome sequencing, molecular docking, Western blotting, and qRT-PCR. The results revealed that SDS might target PPARα to regulate autophagy and apoptosis, enhancing immunity and alleviating inflammation, to exert preventive and therapeutic activities on NASH.

## 2 Materials and methods

### 2.1 Animals and treatment

All animal experimental procedures were approved by the Institutional Animal Care and Use Committee of the Ocean University of China (approval number: OUC-SMP-2024-04-01). Specific pathogen-free male C57BL/6J mice (7 weeks of age) were purchased from Beijing Weitong Lihua Experimental Animal Technology Co., Ltd. (China), housed in a 23°C–25°C environment with a light–dark cycle of 12 h, and given shredded wood flour bedding for social activity.

The mice were randomly divided into four groups: (1) mice of control groups (n = 15) were fed with a controlled diet (complete semisynthetic column diet containing 18% crude proteins and 5% cellulose, following the Chinese Association for Laboratory Animal Sciences); (2) mice of the MCD group (n = 15) were fed an MCD diet (a diet composed of high sucrose (40%) and fat (10%) but without methionine and choline), which is a classical used nutritional model of NASH; (3) a mouse group (n = 15) was fed an MCD diet supplemented with SDS at a dose of 24 mg/kg of body weight continuously for 28 days to study the preventive effect of SDS during NASH formation (MCD-SP); and (4) a NASH mouse model (MCD-induced) group (n = 15) was fed the controlled diet and SDS at a dose of 24 mg/kg of body weight for 28 days to study the therapeutic effect of SDS on NASH (MCD-ST). SDS was dissolved in distilled water (24 mg/mL). An aliquot portion of the SDS solution (correlated with each mouse’s body weight) was administered via gavage. Fresh mineral water in drinking bottles was replaced daily. Throughout the study period, all mice had free access to diet and water. Body weights were measured and recorded every week. At the end of the study, 4 weeks for the control, MCD, and MCD-SP groups or 8 weeks for the MCD-ST group, the mice were fasted overnight and euthanized using pentobarbital. Blood and livers were sampled. Serum samples were centrifuged at 4°C (3,000 rpm, 15 min). The serum and liver (after measuring and photographing) were frozen in liquid nitrogen and stored at −80°C until analysis.

### 2.2 Serum and hepatic biochemical indicators

The serum lipid profiles, including triglyceride (TG), total cholesterol (TC), and liver functional measures of the levels of serum aspartate aminotransferase (AST) and alanine aminotransferase (ALT), were quantified using commercial kits (Nanjing Jiancheng Institute of Bioengineering, China), following the manufacturer’s instructions. The serum concentrations of pro-inflammatory cytokines, such as tumor necrosis factor α (TNF-α), interleukin-2 (IL-2), interleukin-10 (IL-10), and interleukin-17 (IL-17), were determined using sandwich enzyme-linked immunosorbent assay (ELISA) kits (Shanghai Enzyme-linked Biotechnology, China), according to the manufacturer’s instructions. Individual liver tissue samples (10 mg each) were homogenized in 90 μL of anhydrous ethanol in a Potter–Elvehjem tissue homogenizer and centrifuged at 2,500 × g for 10 min to obtain the liver tissue extract. The concentrations of TG and TC in the liver were quantified using commercial kits (Nanjing Jiancheng Institute of Bioengineering, China), following the manufacturer’s instructions.

### 2.3 Histological analysis

Here, 5-μm paraffin-embedded liver sections were stained with hematoxylin and eosin (H&E) staining and Sirius red after fixing in 4% paraformaldehyde at 4°C overnight, which was then embedded in paraffin and dehydrated. Then, 5-μm frozen liver sections were stained with Oil Red O solution in 60% isopropanol after embedding in the optimum cutting temperature compound (Tissue-Tek, Laborimpex). Images were obtained using an optical microscope (Olympus, BX51, Tokyo, Japan). Then, the sum of NAFLD activity scores (NASs) was used to determine the severity of NASH. The NAS system mainly included a semi-quantitative analysis of three pathological features of the liver: lobular inflammation (0–3), hepatic steatosis (0–3), and ballooning (0–2). The sum of the three scores was the NAS. NAS of ≥5 was diagnosed as NASH; NAS between 3 and 4 was NASH suspected; and NAS <3 was not diagnosed as NASH (Han Y. et al., 2023).

For immunohistochemistry (IHC) analysis, the paraffin-embedded liver sections were dewaxed and sequentially incubated with an anti-B-cell lymphoma-2 (BCL-2) primary antibody (1:200; abs131701, Absin, China), anti-BCL-2-Associated X Protein (BAX) primary antibody (1:50; A5131, Selleck, China), and anti-nuclear factor kappa-light-chain-enhancer of activated B cell (NF-κB) primary antibody (1:500; 8242T, CST, China) at 4°C overnight. On the following day, the slides were incubated with a biotinylated secondary antibody (Proteintech, Wuhan, China) at room temperature for 1 h. Positive staining was detected using a 3,3ʹdiaminobenzidine chromogenic reagent, and then, all sections were counterstained with hematoxylin. Immunohistochemistry images were acquired using an optical microscope (Olympus, BX51, Tokyo, Japan).

### 2.4 Transmission electron microscopy

The autophagosomes of liver tissues were observed by transmission electron microscopy (TEM). Parts of the liver were fixed with 1.25% glutaraldehyde for 1 day and then post-fixed in 1% osmium tetroxide for 1 h. Dehydration was done in a concentration gradient of ethanol, followed by propylene oxide. When incubated in 70% ethanol, the pellet was stained embolic with 1% uranyl acetate. Finally, the pellet was embedded in EPON resin. Ultrathin sections were post-stained with uranyl acetate and Reynold’s lead citrate routinely. Electron micrographs were taken using a transmission electron microscope at 80 kV (JEM-1400Flash, Tokyo, Japan).

### 2.5 Quantitative real-time PCR

The total RNA was extracted from the mouse liver tissues using the MolPure^®^ TRIeasy™ Plus Total RNA Kit (Yeasen, Shanghai, China). The cDNA was reverse-transcribed using a FastKing RT SuperMix kit (TIANGEN, Beijing, China), following the manufacturer’s protocol. Furthermore, quantitative real-time PCR (qRT-PCR) was performed using the Bio-Rad Laboratories CFX Connect™ Real-Time PCR Detection System using Fast SYBR Green Master Mix (Yeasen, Shanghai, China). The results were quantified by the 2^−ΔΔCT^ method relative to the housekeeping gene actin. The primer pairs are listed in Table 1.

**TABLE 1:** Forward and reverse primer sequences for qRT-PCR.

Primer	Forward sequence	Reverse sequence
GAPDH	GTG​AAG​GTC​GGT​GTG​AAC​GG	GTG​ATG​GCA​TGG​ACT​GTG​GTC
TNF-α	GCCACCACGCTCTCTCTG	GGTGTGGGTGAGGAGCA
IL-2	AAA​AGC​TTT​CAA​TTG​GAA​GAT​GCT​G	TTG​AGG​GCT​TGT​GTG​AGA​TGA
IL-17	TTT​AAC​TCC​CTT​GGC​GCA​AAA	CTT​TCC​CTC​CGC​ATT​GAC​AC
IL10	GCC​TTA​TCG​GAA​ATG​ATC​CA	AGG​GGA​GAA​ATC​GAT​GAC​AG
p62	AGT​GAT​GAG​GAG​CTG​ACA​ATG​GCT	GCC​AGC​CAA​AGT​GTC​CAT​GTT​TCA
LC3	TAG​GCA​CCC​ACA​TAG​GGT​ATT​A	CTA​CAA​CAC​CAG​ACC​TGC​TTA​G
PPARα	AGA​GCC​CCA​TCT​GTC​CTC​TC	ACT​GGT​AGT​CTG​CAA​AAC​CAA​A

### 2.6 Western blotting

Liver tissues were ground using a grinding machine (KZ-II, Servicebio, Wuhan, CN) and then lysed in a radioimmunoprecipitation assay buffer containing a protease inhibitor (Beyotime, Shanghai, China) to extract the protein. After sodium dodecyl sulfate polyacrylamide gel electrophoresis, the proteins were transferred to 0.45-μm-pore polyvinylidene fluoride membranes (Millipore, MA, United States). After blocking with 5% non-fatty dry milk, the membranes were incubated with primary antibodies against sequestosome-1 (p62) (1:1,000, 5114T, CST, United States), microtubule-associated protein-1 light-chain 3 (LC3) A/B (1:1,000, 12741T, CST, United States), PPARα (1:1,000, 66826-1-Ig, Proteintech, China), and β-actin (1:5,000, 81115-1-RR, Proteintech, China) (Wang et al., 2022). Following the incubation of HRP-labeled goat anti-rabbit IgG (1:5,000, Proteintech, China) or goat anti-mouse IgG (1:5,000, Proteintech, China), the bands were analyzed using the gel documentation system (Bio-Rad, CA, United States).

### 2.7 TdT-mediated dUTP nick-end labeling staining

Apoptosis of liver tissues was detected using a TdT-mediated dUTP nick-end labeling (TUNEL) staining kit (Roche, Basel, Switzerland). In brief, the mouse liver tissues were cut into sections. Next, these sections were deparaffinized. After that, the sections were permeabilized with 0.2% Triton X-100 for 10 min at room temperature and then stained with 50 μM TUNEL reagent for 1 h at 37°C in the dark. Then, 4ʹ-6-diamino-2-phenylindole (Sigma-Aldrich, St. Louis, MO, United States) was used to stain the cell nuclei for 20 min in the dark. Finally, the positive cells were visualized and counted under a fluorescence microscope (Leica, Wetzlar, Germany).

### 2.8 Flow cytometry

Intrahepatic immune cells and spleen immune cells were separated from the perfused liver and spleen by enzyme digestion and density gradient centrifugation. Subsequently, anti-CD4 (67786-1-Ig, Proteintech, China) and anti-IL-17 (66148-1-Ig, Proteintech, China) of T helper 17 (Th17) cells were double-labeled; anti-CD3 (17617-1-AP, Proteintech, China) and anti-CD4 of T helper cells were detected by double-labeling; anti-CD3 and anti-CD8 (sc-53063, Santa, United States) of killer T cells were detected by double-labeling; anti-CD4, anti-CD25 (E-AB-F1194C, Elabscience, China), and anti-Foxp3 (12653S, CST, United States) were detected by regulatory T cells (Tregs); anti-CD16 (E-AB-F1236C, Elabscience, China) and anti-CD56 (14255-1-AP, Proteintech, China) of natural killer (NK) cells were double-labeled; anti-CD11b (66519-1-Ig, Proteintech, China) of macrophages was single-labeled; and anti-CD19 (66298-1-Ig, Thermo Fisher, United States) and anti-CD268 (14-9,117-82, Proteintech, China) of B cells were double-labeled. The cells were added to the cell suspension in PBS for 1 h at room temperature. After washing with PBS three times, a secondary fluorescein isothiocyanate (FITC)-conjugated anti-mouse antibody (E-AB-1088, Elabscience, China) or FITC-conjugated anti-rabbit antibody (E-AB-1111, Elabscience, China) was added for further incubation at 4°C for 1 h in dark. The cells were analyzed by flow cytometry using a BD FACSCalibur instrument. Data were analyzed using FlowJo software (v10.2; FlowJo, LLC, Ashland, OR, United States).

### 2.9 Transcriptomic analysis

Three liver samples in each group, including the MCD, MCD-SP, and MCD-ST groups, were subjected to total RNA extraction using the RNAiso Plus reagent (9109, TaKaRa, Japan). The RNA sample was checked for a RIN to inspect the RNA integrity using the Bioanalyzer 2100 system (Agilent Technologies, Santa Clara, CA, United States). The qualified total RNA was further purified using an RNAClean XP Kit (A63987, Beckman Coulter, Inc., Kraemer Boulevard Brea, CA, United States) and RNase-Free DNase Set (79254, QIAGEN GmBH, Germany). Sequencing libraries were prepared using the VAHTS mRNA-seq v2 Library Prep Kit (Illumina, United States). The concentration and size of libraries were detected using a Qubit^®^ 2.0 Fluorometer (Life Technologies, United States) and the Agilent Bioanalyzer 2100 system, respectively. After cluster generation and first-way sequencing primer hybridization, the cDNA libraries were sequenced on the Illumina NovaSeq 6000 platform (Illumina, United States), and paired-end reads were generated. Raw read data in fastq format were filtered using Seqtk software to remove the adaptor sequence and low-quality sequence reads. Clean reads were mapped to the genome using HISAT2 (v2.0.4). StringTie (v1.3.0) was used to calculate the fragments of each gene after mapping. The quantification of the gene expression level was estimated as fragments per kilobase of exon model per million mapped reads.

### 2.10 Proteomic analysis

Next, 300 μL of 8 M urea was added to the liver tissues, and the protease inhibitor was added at 10% of the lysate for proteomic sequencing. After centrifuging at 14,100 × g for 20 min, the supernatant was collected for protein extraction. The protein concentration was determined using the Bradford method, and the remaining sample was frozen at −80°C.

Next, the protein was digested and desalinated. A 100-µg aliquot of extracted proteins from each sample was then subjected to reduction. The sample was diluted four times by adding 25 mM ammonium bicarbonate buffer after adding 200 mM dithiothreitol solution and incubating at 37°C for 1 h. Then, trypsin was added to the sample (trypsin:protein = 1:50) and incubated at 37°C overnight, and 50 μL 0.1% formic acid (FA) was added to terminate digestion in the next day. Then, 100 μL 100% acetonitrile (ACN) was used to wash the C18 column, and the column was centrifuged at 1,200 rpm for 3 min. The column was washed once with 100 μL of 0.1% FA and centrifuged at 1,200 rpm for 3 min and then transferred to the new EP tubes and centrifuged at 1,200 rpm for 3 min. The column was washed twice with 100 μL of 0.1% FA and centrifuged at 1,200 rpm for 3 min. Then, the column was washed once with 100 μL of pH 10 water and transferred to the new EP tubes and eluted with 70% ACN. The eluents of each sample were combined, lyophilized, and stored at −80°C until loading.

For spectral library generation, samples were fractionated using a high-pH reversed-phase fractionator, as previously described and measured in the data-dependent acquisition (DDA) mode. In brief, the mass spectrometer was operated on a quadrupole Orbitrap mass spectrometer (Q Exactive HF-X, Thermo Fisher Scientific, Bremen, Germany) coupled to an EASY nLC 1200 ultra-high pressure system (Thermo Fisher Scientific) via a nano-electrospray ion source. Then, 500 ng of peptides were loaded on a 25-cm column (150-μm inner diameter, packed using ReproSil-Pur C18-AQ 1.9-µm silica beads). The peptides were separated using a gradient of 8%–12% B in 7 min, then 12%–30% B in 48 min, and stepped up to 40% in 10 min, followed by a 15-min wash at 95% B at 600 mL per minute where solvent A was 0.1% FA in water and solvent B was 80% ACN and 0.1% FA in water. The total duration of the run was 80 min. The column temperature was maintained at 60°C using an in-house-developed oven. In brief, the mass spectrometer was operated in the “top-40” data-dependent mode, collecting MS spectra using the Orbitrap mass analyzer (120,000 resolution, 350–1,500 m/z range) with an automatic gain control (AGC) target of 3E6 and a maximum ion injection time of 80 ms. The most intense ions from the full scan were isolated with an isolation width of 1.6 m/z. Following higher-energy collisional dissociation with a normalized collision energy of 27, the MS/MS spectra were collected in the Orbitrap (15,000 resolution) with an AGC target of 5E4 and a maximum ion injection time of 45 ms. Precursor dynamic exclusion was enabled with a duration of 16 s. For data-independent acquisition (DIA), the acquisition method consisted of one MS1 scan (350–1,500 m/z, resolution 60,000, maximum injection time 50 ms, and AGC target 3E6) and 42 segments at varying isolation windows from 14 m/z to 312 m/z (resolution 30,000, maximum injection time 54 ms, and AGC target 1E6). The stepped normalized collision energy was 25, 27.5, and 30. The default charge state for MS2 was set to 3.

The MS data on the fractionated pools (DDA MS data, six fractions) and the single-shot samples (DIA MS data) were used to generate a DDA library and direct-DIA library, respectively, which were computationally merged into a hybrid library in Spectronaut software (Biognosys, version 15.7.220308.50606). The hybrid spectral library was used to search the MS data on the single-shot samples in Spectronaut software for the final protein identification and quantitation. Carbamidomethylation was used as the fixed modification and acetylation of the protein N-terminus, while the oxidation of methionine was used as variable modifications. Default settings were used for other parameters. In brief, a trypsin/P proteolytic cleavage rule was used, permitting a maximum of two miscleavages and a peptide length of 7–52 amino acids. Protein intensities were normalized using the “Local Normalization” algorithm in Spectronaut based on a local regression model. Spectral library generation stipulated a minimum of three fragments per peptide, and maximally, the six best fragments were included. A protein and precursor false discovery rate of 1% was used, and protein quantities were reported in samples only if the protein passed the filter (“Q-value sparse” mode data filtering).

Gene Ontology (GO) analysis was conducted using the InterProScan-5 program against the non-redundant protein database, and the Kyoto Encyclopedia of Genes and Genomes (KEGG) was used to analyze the protein family and pathway.

### 2.11 Network pharmacology prediction analysis

The SMILES structure of SDS was obtained using the ChemSpider database (http://www.chemspider.com/) and was introduced into the SwissTargetPrediction online target screening platform (http://www.swisstargetprediction.ch/) to obtain the predicted target information on SDS.

The DisGeNET database (http://www.disgenet.org/) was used to obtain targets related to NASH by using “Non-alcoholic steatohepatitis” as a keyword. The obtained relevant targets were imported into the UniProt (https://www.uniprot.org/) database to correct the target information.

The SDS responding targets and the NASH responding targets were intersected in Draw Venn Diagram (http://bioinformatics.psb.ugent.be/webtools/Venn/), and the intersection targets were obtained, which were the SDS targets of treating NASH. The obtained intersection targets were imported into the STRING database protein–protein interaction (PPI) network, where nodes represent target proteins and edges represent interactions between nodes. PPI network information obtained was saved as a TSV file and imported into the network topology attribute analysis software application Cytoscape to build an SDS-NASH target network. Then, the Network Analyzer function in this software program analyzed the network topology to obtain the degree value of each node, which reflected the importance of SDS-NASH targets in the network based on the degree value.

Molecular docking between SDS and the top five target proteins of SDS in the treatment of NASH was verified using AutoDockTools software. The 2D structure of SDS was downloaded from the ChemSpider database and imported into Chem3D software in mol format, and the mechanical structure was optimized to export in mol2 format. The crystal structure of the target protein was selected by the RCSB PDB (http://www.rcsb.org/) database, and its docking active center and free binding energy were calculated using AutoDockTools software. Finally, the docking results were visualized using PyMOL software.

### 2.12 Statistical analysis

Data were presented as the mean ± standard error mean. The comparison between groups was performed by one-way ANOVA and *post hoc* Student’s *t*-test, and the multiple comparisons were analyzed by Tukey’s honestly significant difference test using SPSS software (version 20.0, IBM, Armonk, NY, United States). The statistically significant difference between various groups was considered when a *p*-value of <0.05 was obtained.

## 3 Results

### 3.1 SDS inhibited the MCD-induced NASH formation and exerted promising NASH therapeutic activity *in vivo*

**FIGURE 1:**
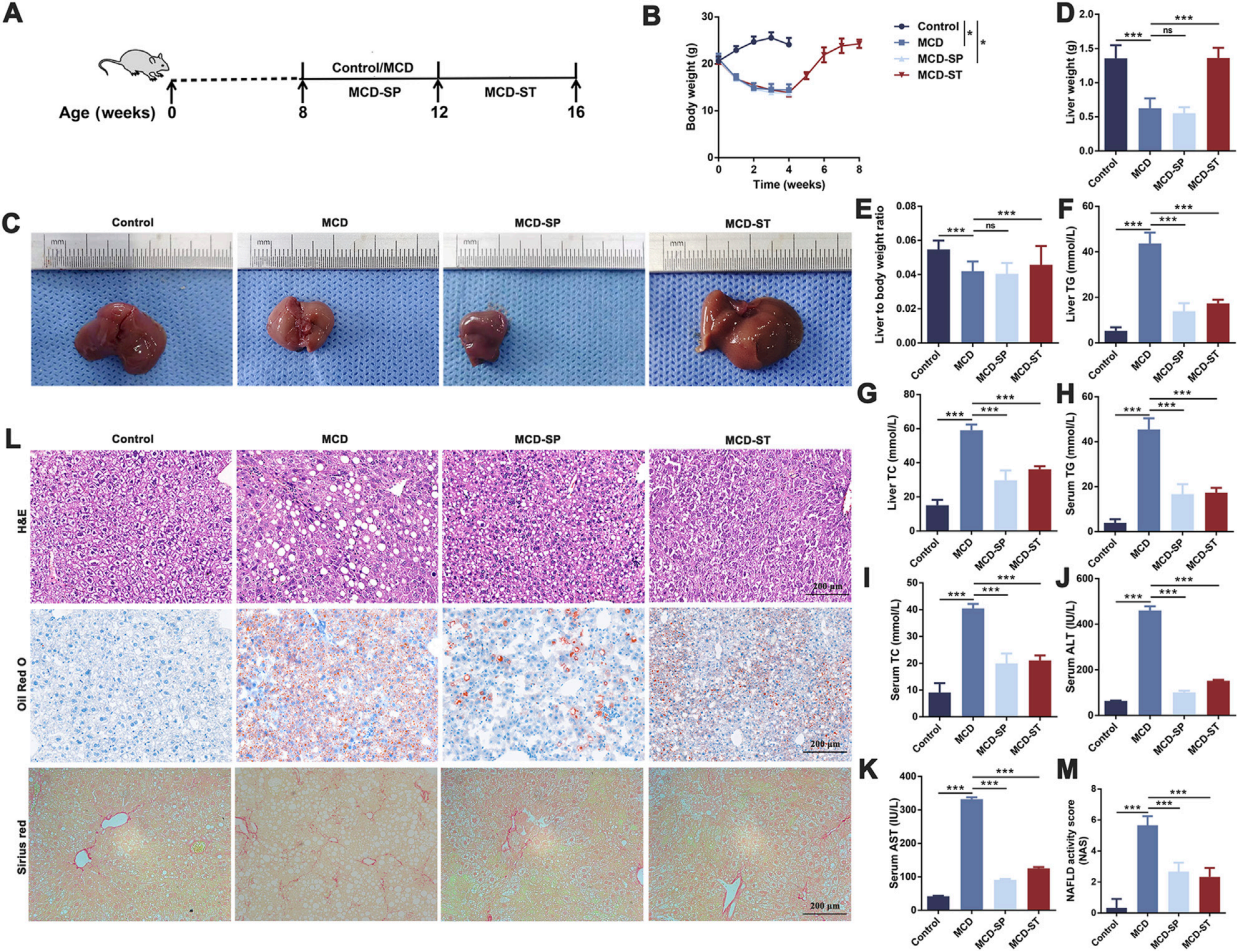
SDS significantly inhibited the formation of NASH and had a significant therapeutic effect on the mouse NASH model. **(A)** Schematic diagram of the control, MCD, MCD-SP, and MCD-ST mouse models. **(B)** Changes in the body weight of mice. **(C)** Image of the mouse liver. **(D)** Mouse liver weight. **(E)** Mouse liver-to-body weight ratio. **(F)** Mouse liver TG. **(G)** Mouse liver TC. **(H)** Mouse serum TG. **(I)** Mouse serum TC. **(J)** Mouse serum TG. **(K)** Mouse serum AST. **(L)** H&E, Oil Red O, and Sirius Red staining of liver sections from the indicated groups of mice. Scale bar = 200 µm. **(M)** NAFLD activity score (NAS). NAS is the sum of the steatosis, inflammation, and ballooning score. Data were representative of one or three independent experiments with n = 6 per group. Data were analyzed by Student’s *t*-test and expressed as the mean ± SEM. *p < 0.05 and ***p < 0.001; ns, no significance. Control group, MCD group, MCD-SP group, and MCD-ST group. Control, normal diet-fed mice; MCD, MCD-fed mice; MCD-SP, MCD with 24 mg/kg/d of SDS; MCD-ST, NASH model mice were fed with the control diet with 24 mg/kg/d of SDS.

The MCD diet is a classic and useful method to induce NAFLD/NASH in rodents, which was commonly used for the pathogenesis, prevention, and therapy investigation (Parlati et al., 2021). In this study, the positive function of SDS in the progression of NASH was explored using this mouse model. In detail, 7-week-old male C57BL/6J mice were fed with an MCD diet for 4 weeks to develop NASH (Figure 1A). As shown in Figure 1B, notable weight loss was observed in the MCD diet group mice compared to the control group, which was from the beginning of the diet until euthanasia. Macroscopic observation showed a significant size decrease and yellow color change in the livers of the MCD diet group (Figure 1C). The liver weights of the MCD group mice were significantly decreased compared with the control group (Figure 1D). Furthermore, the liver-to-body weight ratio also confirmed this conclusion (Figure 1E). Blood biochemistry examination revealed a significant upregulation both in the serum and liver tissue levels of TC and TG, as well as the levels of serum ALT and AST in the MCD group compared to these biochemical markers in the control group (Figures 1F–K). The liver histological changes in the MCD group, including hepatic steatosis, ballooning, inflammation, and fibrosis, are the typical pathological findings of human NASH (Tincopa and Loomba, 2023). Specifically, H&E and oil red O staining presented a noticeable increase in the accumulation of lipid droplets, hepatocyte ballooning, and inflammatory cell infiltration in MCD group liver sections, while Sirius red staining revealed significantly increased hepatic fibrosis (Figure 1L). Additionally, the NAS of the MCD group was >5 (Figure 1M). These results demonstrated that the MCD-induced NASH model presented the major features of human NASH, which could be used to study the pathogenesis and therapeutic of NASH.

To explore the potential clinical prevention value of SDS on hepatic steatosis and liver injury, oral administration of SDS (24 mg/kg/day) was supplied accompanied by an MCD diet for 4 weeks (Figure 1A). Compared with the MCD group, SDS treatment had no effect on body weight, liver size, liver weight, and liver to body weight ratio in MCD-SP group mice (Figures 1B–E), but H&E and Oil Red O staining presented that SDS intake remarkably prevented MCD-induced hepatic lipid droplets, hepatocyte ballooning, and inflammatory cell infiltration, and the NAS was significantly reduced in the MCD-SP group (Figures 1L, M). Moreover, SDS treatment sharply decreased serum TG, TC, ALT, and AST levels, as well as hepatic TG and TC contents in the MCD-SP group, which contrasted with the MCD group (Figures 1F–K). Furthermore, SDS treatment crucially prevented MCD-induced liver fibrosis, as shown by the result of Sirius Red staining (Figure 1L). These results indicated that SDS significantly inhibited NASH formation.

To validate the therapeutic effect of SDS on NASH *in vivo*, we used the mice in the MCD-ST group to study the therapeutic effect of SDS on hepatic steatosis and liver injury. In detail, MCD diet-induced NASH mice were fed a controlled diet with the oral administration of SDS (24 mg/kg/d) continuously for 4 weeks (Figure 1A). As shown in Figures 1B, C, the weight of mice in the MCD-ST group increased significantly once receiving SDS treatment, which almost recovered to the same level as that in the control group in 28 days of SDS administration. Compared with the MCD group, there was no significant difference in liver weight between the MCD-ST group and the control group mice (Figure 1D). The results were consistent with the liver weight-to-body weight ratio (Figure 1E). Compared with the MCD group, the contents of TG and TC in the liver and TG, TC, ALT, and AST in serum were also significantly decreased in the MCD-ST group (Figures 1F–K). Furthermore, H&E and Oil Red O staining presented a notable decrease in the accumulation of lipid droplets, hepatocyte ballooning, and inflammatory cell infiltration in MCD-ST mouse liver sections, while Sirius Red staining revealed significantly decreased hepatic fibrosis (Figure 1L). In addition, it was worth noting that the NAS of the MCD-ST group was <3, indicating that the mice in this group were not diagnosed with NASH. Taken together, these results suggested that SDS exerted a significant therapeutic function in NASH mice.

### 3.2 SDS treatment upregulated liver autophagy and downregulated liver apoptosis

**FIGURE 2:**
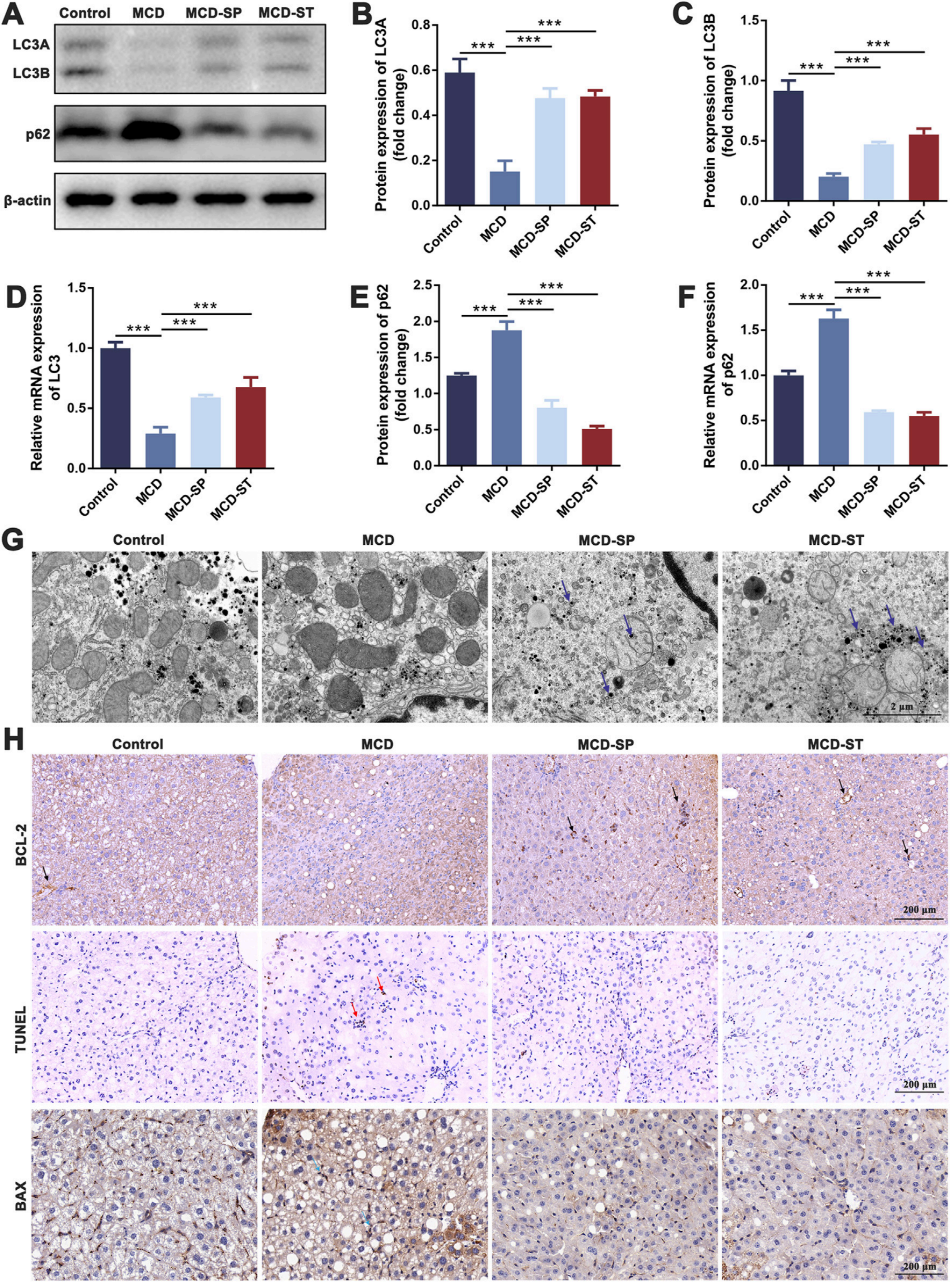
SDS significantly upregulates liver autophagy and downregulates liver apoptosis in NASH mice. **(A)** Western blotting of LC3A/B and p62 in the liver of the mice. **(B, C, E)** The relative levels of LC3A, LC3B, and p62 in the liver of the mice were analyzed by Western blotting. **(D, F)** qRT-PCR analyses of the relative expressions of LC3 and p62 in the liver of the mice. **(G)** Mouse liver autophagosome observed by TEM. The blue arrow indicates the autophagosome. Scale bar = 2 µm. **(H)** IHC staining of BCL-2 and BAX and TUNEL staining of liver sections from the indicated groups of mice. The black arrow indicates inhibition of apoptosis in the mouse liver. The red arrow indicates apoptosis of the mouse liver. The blue arrow indicates the promotion of apoptosis in the mouse liver. Scale bar = 200 µm. Data were representative of one or three independent experiments with n = 6 per group. Data were analyzed by Student’s *t-*test and expressed as the mean ± SEM. ***p < 0.001. Control group, MCD group, MCD-SP and MCD-ST group. Control, normal diet-fed mice; MCD, MCD-fed mice; MCD-SP, MCD with 24 mg/kg/d of SDS; MCD-ST, NASH model mice were fed the control diet with 24 mg/kg/d of SDS.

Programmed cell death, such as autophagy and apoptosis, plays a key role in the development of NASH (Shojaie et al., 2020). Therefore, we first evaluated the effect of SDS on autophagy response. We detected the expression of autophagy marker LC3 in mouse liver tissues by qRT-PCR and Western blotting in these four groups of mice. The results showed that LC3 expression was significantly decreased in the MCD group compared with the control group, while MCD-SP and MCD-ST remarkably reversed this situation (Figures 2A–D). In addition, the p62 expression level was negatively correlated with autophagy activity and can also be used to monitor autophagy flux. The qRT-PCR and Western blotting results showed that the expression of p62 was downregulated in the MCD-SP and MCD-ST groups compared with the MCD group (Figures 2A, E, F). Next, the formation of autophagosomes in liver cells was observed by TEM. As shown in Figure 2G, the results showed that the MCD group formed fewer autophagosomes, while the number of autophagosomes in the liver cells of mice in the MCS-SP and MCS-ST groups was significantly increased, indicating that SDS could significantly upregulate liver autophagy induced by the MCD diet.

Apoptosis is an important biological process that plays a crucial role in cell fate and homeostasis. Hepatocyte apoptosis is a well-defined form of cell death in NASH and is believed to be a major cause of liver inflammation (Zhao et al., 2020). Therefore, we investigated whether SDS could reduce the level of liver apoptosis in MCD mice using BCL-2 and BAX as the marker of apoptosis. Upon daily intake of SDS, the levels of BCL-2 of liver tissues from MCD-SP and MCD-ST groups significantly recovered to a normal level (Figure 2H). Meanwhile, IHC results of the pro-apoptotic marker BAX also confirmed that compared with the MCD group, SDS could suppress the level of BAX in the MCD-SP and MCD-ST groups (Figure 2H). In addition, we used TUNEL staining to detect the DNA breaks formed when DNA fragmentation occurred in the last phase of liver apoptosis. The TUNEL experiment showed that, compared with the control group, the positive area representing apoptotic cells in the liver tissue of mice in the MCD group was significantly larger, while the positive areas in both the MCD-SP and MCD-ST groups were not significantly changed (Figure 2H). Overall, our results showed the ability of SDS to upregulate liver autophagy and downregulate apoptosis of the liver in the NASH mice.

### 3.3 SDS treatment regulated innate and adaptive immune responses to alleviate inflammation

**FIGURE 3:**
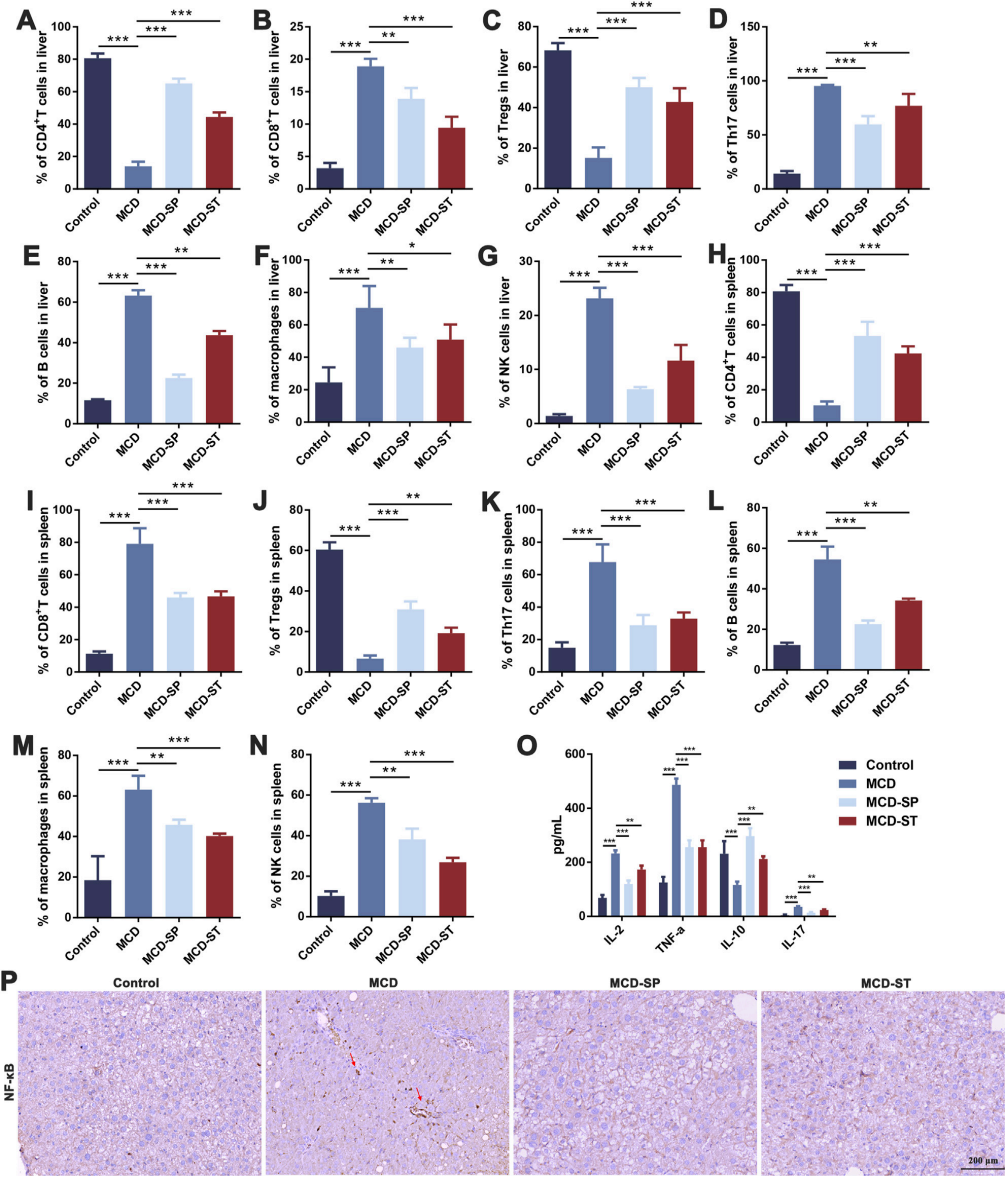
SDS significantly balances intrahepatic immunity and improves inflammatory response in NASH mice. **(A–G)** Frequency of CD4^+^ T cells, CD8^+^ T cells, Tregs, Th17 cells, B cells, macrophages, and NK cells in the mouse liver. **(H–N)** The frequency of CD4^+^ T cells, CD8^+^ T cells, Tregs, Th17 cells, B cells, macrophages, and NK cells in the mouse spleen. **(O)** ELISA of IL-2, TNF-α, IL-10, and IL-17 in mouse serum inflammatory cytokines. **(P)** IHC staining of NF-κB of liver sections from the indicated groups of mice. The red arrow indicates inflammation of the mouse liver. Scale bar = 200 µm. Data were representative of one or three independent experiments with n = 6 per group. Data were analyzed by Student’s *t*-test and expressed as the mean ± SEM. *p < 0.05, **p < 0.01, and ***p < 0.001. Control group, MCD group, MCD-SP and MCD-ST group. Control, normal diet-fed mice; MCD, MCD-fed mice; MCD-SP, MCD with 24 mg/kg/d of SDS; MCD-ST, NASH model mice were fed the control diet with 24 mg/kg/d of SDS.

The hepatic immune cells were reshaped during NASH, which involved the inflammatory processes that triggered liver injury, fibrosis, and evolution toward cirrhosis and hepatocellular carcinoma. Both innate immune and adaptive immunity contribute to NASH-associated inflammation (Zhou et al., 2022). To determine how SDS regulated immune responses to influence the progression of MCD-induced NASH, the liver and spleen were collected to detect immune-related indexes after the mice were euthanized. The frequencies of adapted immune CD4^+^ T cells, CD8^+^ T cells, Tregs, Th17 cells, and B cells, as well as innate immune macrophages and NK cells in mouse livers, were first measured by flow cytometry. Compared with the control group, the frequencies of CD8^+^ T cells, Th17 cells, B cells, macrophages, and NK cells in the MCD group were significantly upregulated, while the frequency of CD4^+^ T cells and Tregs was significantly downregulated. The above condition could be reversed in the MCD-SP and MCD-ST groups treated with SDS (Figures 3A–G).

To evaluate whether SDS treatment mediated systemic immune responses, spleen lymphocytes were analyzed by flow cytometry. The MCD group significantly increased the frequency of CD8^+^ T cells, Th17 cells, B cells, macrophages, and NK cells, while it decreased the frequency of CD4^+^ T cells and Tregs. SDS treatment showed a marked reversal effect to MCD-induced changes of spleen lymphocytes (Figures 3H–N). This was consistent with the results of liver immune-related indexes, indicating that SDS treatment had a significant regulatory effect on the immune responses in NASH mice.

Immune cells in the inflammatory responses secrete various cytokines, and at the same time, cytokines also act on the immune cells. Then, the adapted immune cells will produce more cytokines, thus forming a positive feedback regulation (Duan et al., 2022). Therefore, after evaluating the immune cells mentioned above, we subsequently examined the expression levels of inflammatory factors including IL-2, TNF-α, IL-10, and IL-17 in the mouse serum of each group. An ELISA experiment showed that compared with the control group, the levels of IL-2, TNF-α, and IL-17 in the serum of MCD group mice were significantly upregulated, while the level of IL-10 was significantly downregulated. In contrast, the expression levels of IL-2, TNF-α, and IL-17 in MCD-SP and MCD-ST groups were significantly decreased, and the contents of IL-10 were significantly increased (Figure 3O). The IHC results revealed that the NF-κB protein level was significantly increased in the MCD group compared to the control group but decreased in the MCD-SP and MCD-ST groups (Figure 3P). These results suggested that SDS treatment might play a role in rebalancing immunity and inflammatory response.

### 3.4 RNA-seq transcriptomic and label-free quantitative proteomic analysis of mice

**FIGURE 4:**
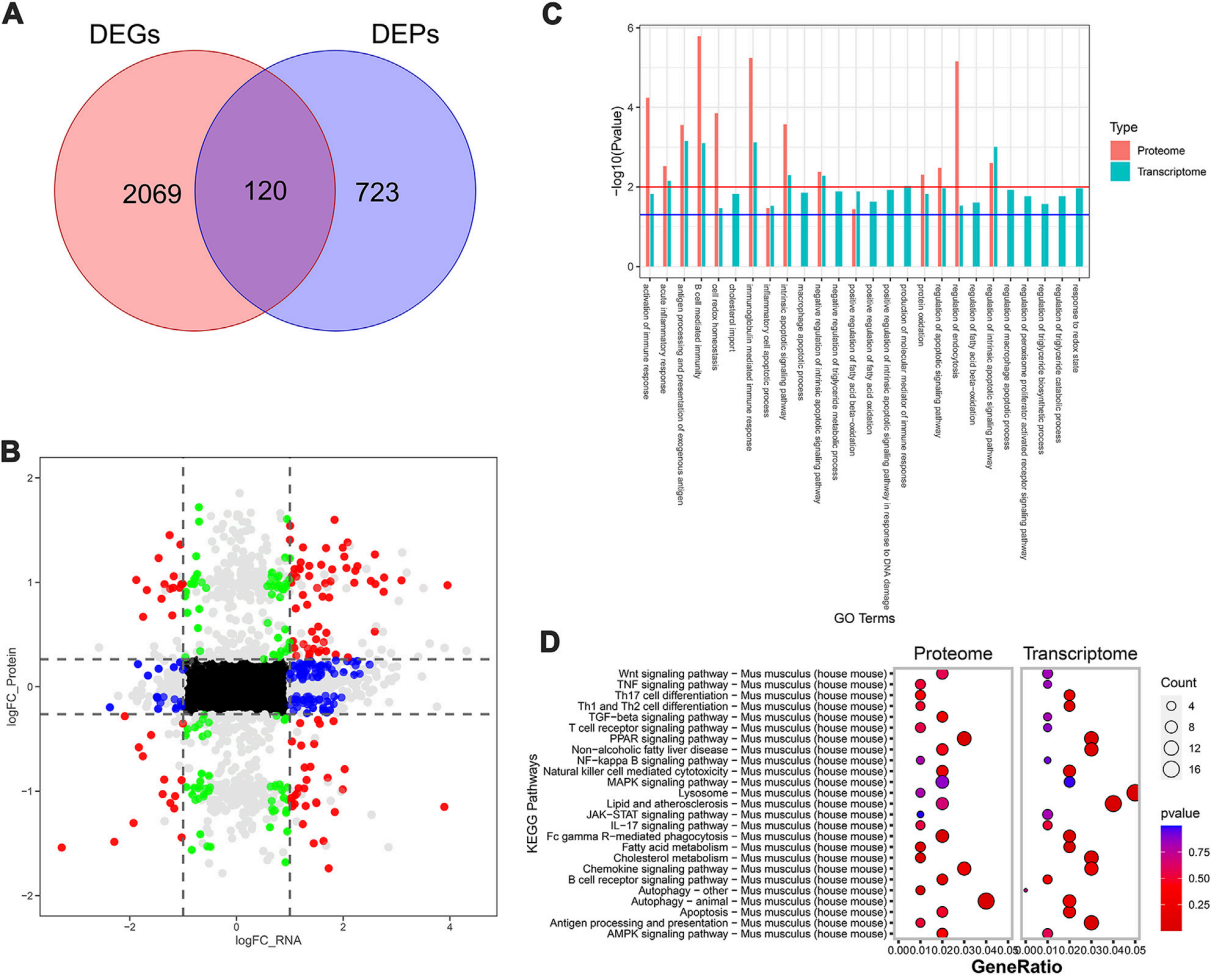
Transcriptomic and proteomic analysis in the MCD group and MCD-SP group. **(A)** Venn diagram of DEGs and DEPs in the MCD group and MCD-SP group. **(B)** Transcription protein nine-quadrant diagram of DEPs in the MCD group and MCD-SP group. **(C)** GO enrichment pathway column chart of the MCD group and MCD-SP group. Blue columns represent the genes in the transcriptome, and red columns represent the proteins in the proteome. **(D)** KEGG enrichment top 25 pathways bubble map of the MCD group and MCD-SP group. The size of each circle represents the number of significant DEGs and DEPs enriched in the corresponding pathways. The chromatogram from blue to red represents the *p*-value. Data were representative of one or three independent experiments with n = 3 per group. The topological method adopts relativism-betweenness centrality. Data were analyzed by Student’s *t*-test and expressed as the mean ± SEM. The DEGs and DEPs. DEGs, differentially expressed genes; DEPs, differentially expressed proteins.

To further systematically elucidate the molecular mechanism by which SDS inhibited the progression of NASH, we performed RNA-seq-based transcriptomics and MS-based marker-free proteomic sequencing on the livers of three mice in each of the MCD, MCD-SP, and MCD-ST groups (NCBI BioProjects: PRJNA1100189). The results of the comparison between the MCD and MCD-SP groups showed a total of 2,189 differentially expressed genes (DEGs) in the transcriptome dataset and 843 differentially expressed proteins (DEPs) in the proteome, of which 120 proteins were shared by the transcriptome and proteome data (Figures 4A, B). Apart from lipid metabolism-related processes or signaling pathways, both Go functional annotation and KEGG pathway analysis indicated that these 120 proteins were also significantly enriched in autophagy, apoptosis, immunity, and inflammation-related processes or signaling pathways (Figures 4C, D). In addition, the Wnt, TGF-β, MAPK, and PPAR signaling pathways were also significantly enriched in the KEGG pathway analysis (Figure 4D).

**FIGURE 5:**
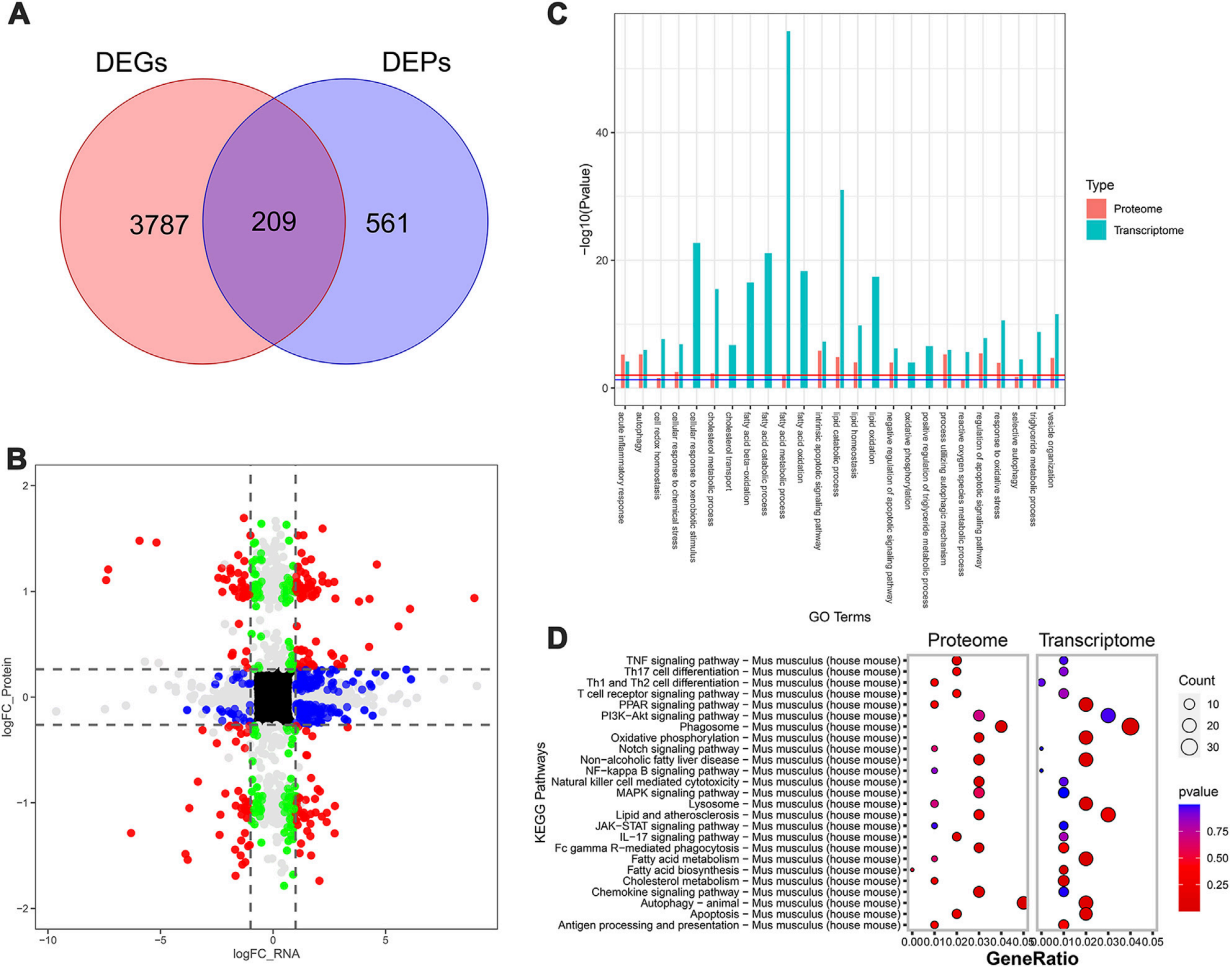
Transcriptomic and proteomic analysis in the MCD and MCD-ST groups. **(A)** Venn diagram of DEGs and DEPs in the MCD group and MCD-ST group. **(B)** Transcription protein nine-quadrant diagram of DEPs in the MCD group and MCD-ST group. **(C)** GO enrichment pathway column chart of the MCD group and MCD-ST group. Blue columns represent the genes in the transcriptome, and red columns represent the proteins in the proteome. **(D)** KEGG enrichment top 25 pathways, bubble map of the MCD group and MCD-ST group. The size of each circle represents the number of significant DEGs and DEPs enriched in the corresponding pathways. The chromatogram from blue to red represents the *p*-value. Data were representative of one or three independent experiments with n = 3 per group. The topological method adopts relativism-betweenness centrality. Data were analyzed by Student’s *t*-test and expressed as the mean ± SEM. The DEGs and DEPs. DEGs, differentially expressed genes; DEPs, differentially expressed proteins.

Next, the MCD and MCD-ST group results showed that 3,996 DEGs and 770 DEPs of the proteome were found in the transcriptome dataset, respectively, of which 209 proteins were shared by the transcriptome and proteome data (Figures 5A, B). Similarly, based on the annotation of the GO database, this overlap between DEGs and DEPs was significantly enriched in the processes related to lipid metabolism, autophagy, apoptosis, and inflammation (Figure 5C). A total of 321 pathways in the SDS treatment of NASH were identified through the KEGG enrichment analysis. Among these pathways, lipid metabolism, autophagy, inflammation, and immune-related signaling pathways were significantly enriched. In addition, the PI3K-AKT, MAPK, and PPAR signaling pathways were significantly enriched (Figure 5D). Notably, the MAPK and PPAR signaling pathways, which participated in the regulatory processes in the MCD-ST group, were also detected to play a significant role in the MCD-SP group. These two signaling pathways have been reported to regulate lipid accumulation, autophagy, apoptosis, immunity, and inflammation in the process of NASH in previous studies. Therefore, based on the above transcriptomic and proteomic analysis results, we speculated that SDS might play a role by regulating MAPK and/or PPAR signaling pathways in NASH mice.

### 3.5 The multiple anti-NASH effects of SDS may target PPARα

**FIGURE 6:**
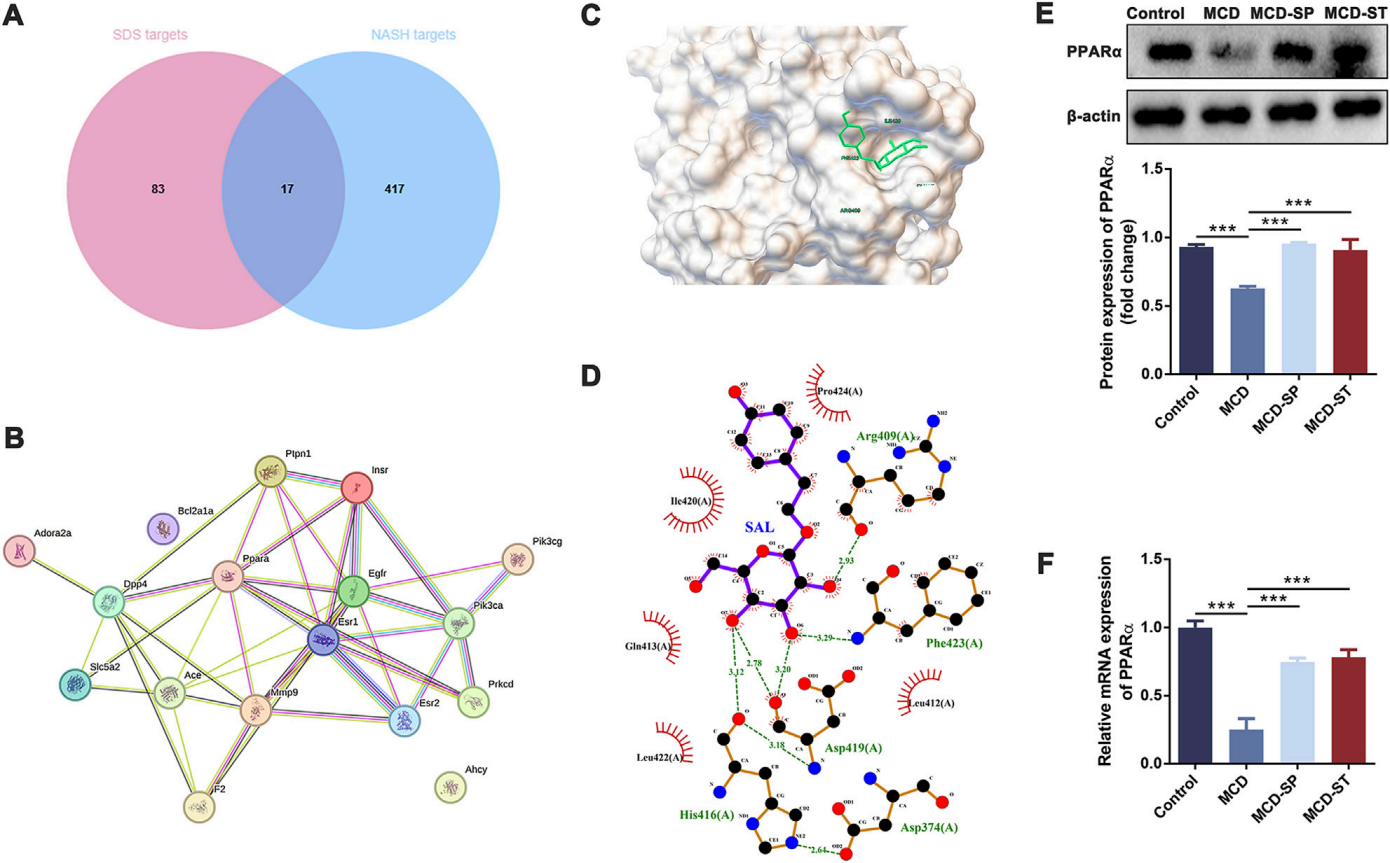
SDS may play a role in the prevention and therapy of NASH through PPARα. **(A)** Venn diagram of SDS targets and NASH targets. **(B)** PPI of intersection targets. **(C, D)** Molecular docking of SDS and PPARα. **(E)** Western blotting of PPARα in the liver of the mice. The relative levels of PPARα in the liver of the mice were analyzed. **(F)** qRT-PCR analyses of the relative expressions of PPARα in the liver of the mice. Data were analyzed by Student’s *t*-test and expressed as the mean ± SEM. ***p < 0.001. Control group, MCD group, and MCD-SP and MCD-ST group. Control, normal diet-fed mice; MCD, MCD-fed mice; MCD-SP, MCD with 24 mg/kg/d of SDS; MCD-ST, NASH model mice were fed with the control diet with 24 mg/kg/d of SDS.

Then, network pharmacology and molecular docking analysis were performed to further confirm the possible role of SDS in preventive and therapeutic activities on NASH. As the results of network pharmacology analysis, 100 SDS-related target genes and 434 NASH-related target genes were obtained. Next, 17 targets of SDS for treating NASH were obtained by plotting Venn diagrams as potential targets (Figure 6A). The 17 targets were imported into the STRING database to obtain protein interaction data, which were imported into Cytoscape software to construct the PPI network (Figure 6B). Topological analysis of the PPI network based on the cyto-NAC function in Cytoscape software identified the top five target genes ranked in the degree of connectivity, namely, EGFR, PPARα, ESR1, ACE, and INSR, which were recognized as key targets for the SDS treatment of NASH. Notably, PPARα is an important component in the PPAR signaling pathway, which has also been shown to play a significant role in the treatment of NASH with SDS in transcriptome and proteomic analysis. In order to evaluate the reliability of the interaction between SDS and PPARα, molecular docking was performed. The minimum binding energy and binding position of SDS and PPARα molecular docking results are shown in Figures 6C, D, which confirmed that SDS and PPARα have binding potential. The minimum binding energy and binding position of SDS and the other four proteins, including EGFR ESR1, ACE, and INSR, are shown in Supplementary Figure S1. Finally, the PPARα expression was verified through the qRT-PCR and Western blot experiments, which showed that PPARα expression was sharply decreased in the MCD group compared with the control group, while MCD-SP and MCD-ST significantly reversed this situation (Figures 6E, F). These findings indicated that SDS might target PPARα to exert preventive and therapeutic activities on NASH.

## 4 Discussion

Due to the increasing incidence and severe consequences of NASH, patients with NASH have become a major population considered for liver transplantation. The limitation to therapy options aggravates this economic burden, so there is an urgent need to develop effective treatments (Lonardo et al., 2022; Mantovani et al., 2020). SDS is the main biological component extracted from *R. rosea* and has a wide range of biological activities, such as lipid-lowering, immune-balancing, anti-oxidation, anti-aging, anti-inflammation, and anti-cancer effects, both *in vivo* and *in vitro* (Zhang et al., 2021). SDS can improve the NAFLD/NASH status by improving abnormal lipid metabolism, inhibiting oxidative stress, regulating apoptosis and autophagy, alleviating inflammatory reactions, reducing fibrosis, and regulating intestinal flora (Qu et al., 2022).

**FIGURE 7:**
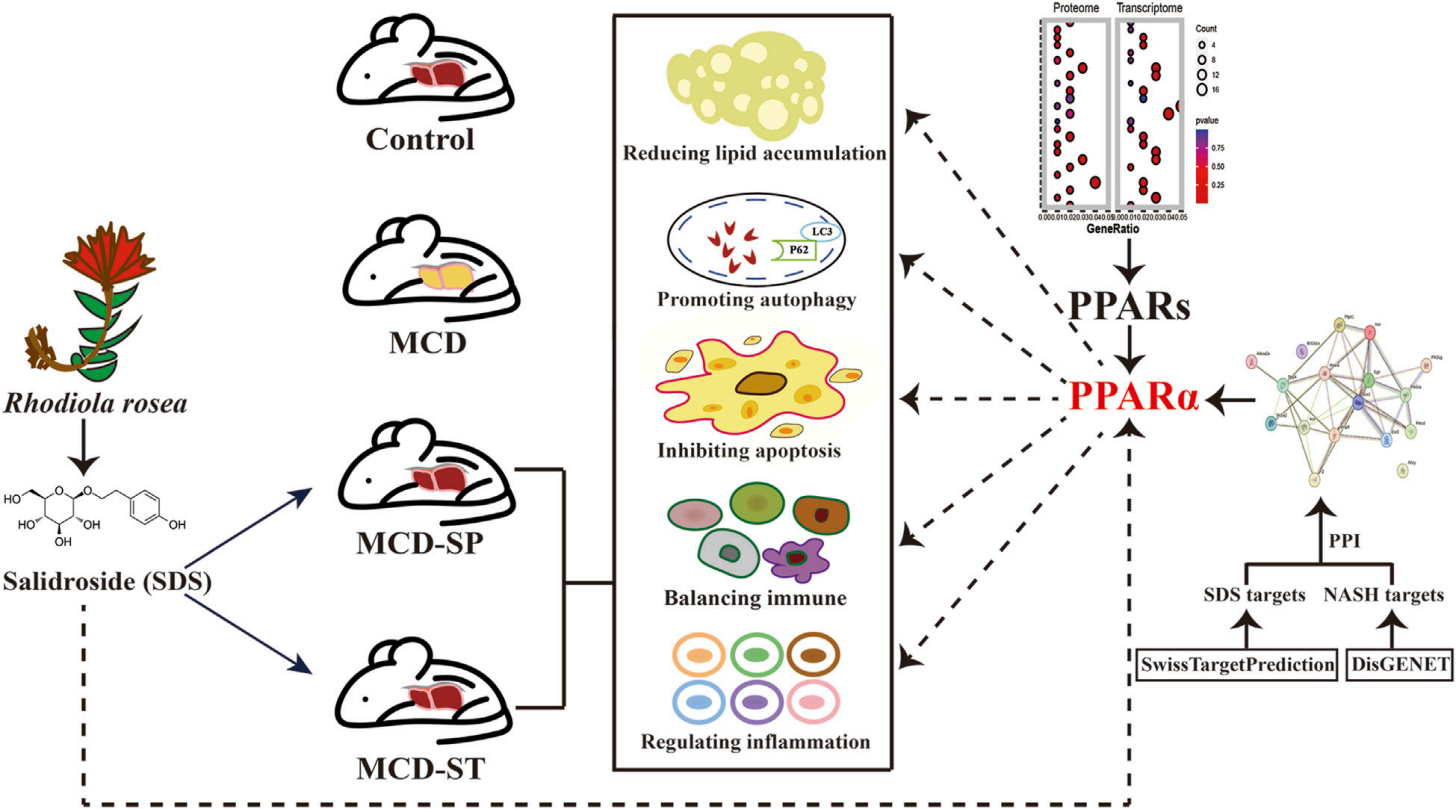
Schematic diagram of the mechanism of SDS improving MCD-induced NASH in mice through PPARα.

In this study, we first comprehensively assessed and confirmed the preventive and therapeutic effects of the oral intake of SDS on NASH (Figure 7). Specifically, although the body weight, liver weight, and liver-to-body weight ratio did not recover in the MCD-SP group, serum and liver biochemical indexes (TG, TC, ALT, and AST) were improved. Comparatively, the MCD-ST group not only reversed the above biochemical indexes but also improved the body weight, liver weight, and liver-to-body weight ratio. Liver pathological sections (H&E, Oil Red O staining and Sirius Red staining) of MCD-SP and MCD-ST mice also thoroughly illustrated that SDS could significantly inhibit the formation of NASH and improve the liver injury of NASH mice. Previous studies revealed that the progression of NASH is closely associated with the processes of autophagy and apoptosis. Autophagy is involved in lipid metabolism and liver injury in NASH, while dysregulated autophagy has also been found to contribute to the development of NASH to liver fibrosis, cirrhosis, and liver cancer (Wu et al., 2018). Apoptosis is an important mechanism contributing to the progression of NASH (Zhao et al., 2024), and the ensuing responses in NASH progression such as cell repair, inflammation, regeneration, and fibrosis may all be triggered by the apoptosis of adjacent cells. In our research, mRNA and protein levels of autophagy markers LC3 and p62 were detected in four groups of mice, and autophagosome formation was observed by TEM, which confirmed that SDS could upregulate liver autophagy. The IHC results of BCL-2, BAX, and TUNEL staining confirmed that SDS could also significantly reduce apoptosis of the mouse liver.

Studies have shown that immune cells and inflammatory factors play important roles in the occurrence and progression of NASH (Peiseler et al., 2022), and SDS was reported to have a good immunomodulatory effect (Yang et al., 2023). The immune system includes adaptive immunity (including CD4^+^ T cells, CD8^+^ T cells, Tregs, Th17 cells, and B cells) and innate immunity (including macrophages and NK cells). In adaptive immunity, CD4^+^ T cells and Tregs are the key regulators of pro-inflammatory and anti-inflammatory immune processes (Woestemeier et al., 2023; Savage et al., 2024). CD8^+^ T cells are involved in the progression of NASH and liver fibrosis, and the reduction in CD8^+^ T cells can prevent the progression of NASH and reduce fibrosis. Furthermore, CD8^+^ T cells can also induce the activation of the NF-κB signaling pathway in hepatocytes and regulate the inflammatory process (Li et al., 2022). Th17 cells secrete IL-17, which can aggravate hepatic steatosis and inflammation and induce the transition from simple steatosis to hepatic steatosis (Zi et al., 2022). In addition, it is reported that there is a large number of B cells in the liver of NASH patients, and these patients have high lobular inflammation and fibrosis, which indicates that B cells may change the course of the disease (Barrow et al., 2021). In innate immunity, macrophages have M1 polarization in NASH and interact with hepatocytes to promote the secretion of inflammatory factors and upregulate the fat synthesis factors, oxidative stress, and endoplasmic reticulum stress, which leads to further deterioration of NASH (Kazankov et al., 2019). Moreover, NK cells play a key role in NAFLD and NASH and can well-coordinate immune responses and regulate inflammation, making it a highly relevant cell type studied under inflammatory conditions (Wang et al., 2021). In this study, for the first time, we evaluated the conditions of adaptive immunity and innate immunity of the mouse liver and spleen to explore how SDS regulated the immune system during the formation of NASH or in NASH patients. Our results revealed that compared with the control group, the frequencies of CD8^+^ T cells, Th17 cells, B cells, macrophages, and NK cells in the MCD group were significantly increased, while the frequencies of CD4^+^ T cells and Tregs were significantly reduced. However, in the MCD-SP and MCD-ST groups treated with SDS, the above situation could be reversed, indicating that SDS treatment had a significant regulatory effect on the immune responses in NASH mice.

Under normal circumstances, cytokines such as ILs and TNF-α play roles in regulating and coordinating immune responses in the immune system. Pro-inflammatory cytokines contribute to the occurrence and spread of autoimmune inflammation, while anti-inflammatory factors contribute to the regression of inflammation and the recovery from autoimmune diseases during the acute stage. When the disease occurs, the equilibrium state is broken, which changes the levels of various inflammatory factors (Peiseler et al., 2022). The ELISA results showed that SDS could inhibit the expressions of IL-2, TNF-α, and IL-17 and increase the expression of IL-10. Moreover, the activation of NF-κB can induce liver inflammation, aggravate fatty toxicity, and lead to the activation of hepatic stellate cells, thus further promoting liver fibrosis (Deng et al., 2022). The results of the IHC experiment showed that compared with the control group, the content of the NF-κB protein in the liver tissue of mice in the MCD group significantly increased, while the content of the NF-κB protein in the liver tissue of mice in the MCD-SP group and MCD-ST group decreased. Thus, these results suggested that SDS might play a significant role in regulating inflammatory responses.

Consequently, in order to determine the molecular mechanism of the anti-NASH effect of SDS mentioned above, we conducted a comparative analysis of the transcriptome and proteome of mouse livers in the MCD and MCD-SP groups and MCD and MCD-ST groups, respectively. The GO and KEGG enrichment analyses showed that the pathways that play a major role in the anti-NASH activity of SDS were mainly concentrated in autophagy, apoptosis, immunity, and inflammation. In addition, the MAPK and PPAR signaling pathways were also significantly enriched. The MAPK signaling pathway has been reported to improve lipid accumulation (Liu et al., 2019), autophagy (Vargas-Pozada et al., 2022), and inflammation (Afrin et al., 2017) in NASH mice. Moreover, it is worth noting that PPAR is involved in the regulation of lipid metabolism (Gross et al., 2017), autophagy (Liang et al., 2023), apoptosis (Ren et al., 2017), immunity, and inflammation (Ni et al., 2022) in the process of NASH, and it is a potentially effective target for the treatment of NASH. Based on this, we speculated that SDS might exert its effects by regulating the MAPK and/or PPAR signaling pathways in NASH mice.

To further elucidate the target of SDS, SDS targets and NASH targets were intersected by Venn diagrams, and PPI analysis was performed. PPI is a systematic analysis of the interaction between a large number of proteins in biological systems, which is of great significance for understanding the working principle of proteins in biological systems, the reaction mechanism of biological signals and energy and material metabolism under special physiological states such as diseases, and the functional links between proteins. As a result, 17 targets of SDS for treating NASH were obtained by plotting Venn diagrams. Subsequently, the top five target genes were identified based on PPI network analysis. As expected, PPARα in the top five genes, which belongs to the PPAR signaling pathway, was shown to play a significant role in the treatment of NASH with SDS treatment. In order to verify the accuracy of the predicted target, the docking analysis between SDS and PPARα was verified through AutoDockTools software. The minimum binding energy and binding position of SDS and PPARα molecular docking results suggested that SDS can play a role in upregulating PPARα in the prevention and therapy of NASH. As reported, the PPAR family is a member of a nuclear receptor superfamily, including PPARα, PPARβ/δ, and PPARγ. PPARα is mainly expressed in the liver, heart, skeletal muscle, brown adipose tissue, intestine, and kidney and promotes energy consumption. PPARα mediates its function by influencing fatty acid transport, esterification, and oxidation. PPARβ/δ is not only widely expressed and participates in fatty acid oxidation but also plays a role in regulating blood glucose levels. In contrast, PPARγ mainly stores energy by promoting lipogenesis and lipid synthesis and shows the highest expression level in white adipose tissue (Christofides et al., 2021). In clinical applications, lanifibranor, an oral PPAR agonist, can activate three PPARs in a balanced way, thus causing beneficial anti-inflammatory, anti-fibrosis, and other vascular and metabolic changes (Francque et al., 2021). At present, it is being analyzed in a global multi-center phase III clinical trial. Lastly, the mRNA and protein levels of PPARα were tested in four groups of mice by experiments, which supported that SDS may regulate liver autophagy, apoptosis, and liver immune environment through the protein of PPARα. To the best of our knowledge, this is the first study to report that the daily oral intake of SDS possesses preventive and therapeutic effects in the NASH formation process or NASH, providing new ideas that SDS could be an ideal candidate drug for the prevention and treatment of NASH.

There were some limitations to this study. The MCD diet used in the current study is considered the most established rodent model of NASH, which mimics classical histopathological features of liver steatosis, apoptosis, oxidative stress, inflammation, and fibrosis, similar to human NASH (Fang et al., 2022; Gallage et al., 2022). However, the model doses do not fully reflect all human NASH characteristics. Mice fed the MCD diet lost weight rather than obesity and lacked insulin resistance, which is commonly observed in patients with NASH (Alshawsh et al., 2022). Thus, in future high-fat and high-fructose models, SDS may play different roles in steatosis, oxidative stress, inflammation, and insulin resistance in obesity-induced 16-week NASH models.

## 5 Conclusion

The results revealed that SDS may exert preventive and therapeutic activities on NASH by regulating autophagy and apoptosis, enhancing immunity, and alleviating inflammation through targeting the protein of PPARα.

## Data Availability

The data presented in the study are deposited in the NCBI BioProject repository, accession number PRJNA1100189.
