# Supplementary figures and images for: Salidroside may target PPARα to exert preventive and therapeutic activities on NASH
Source: Front Pharmacol. 2024 Oct 2;15:1433076. doi: 10.3389/fphar.2024.1433076 (PMC11479876; doi:10.3389/fphar.2024.1433076)

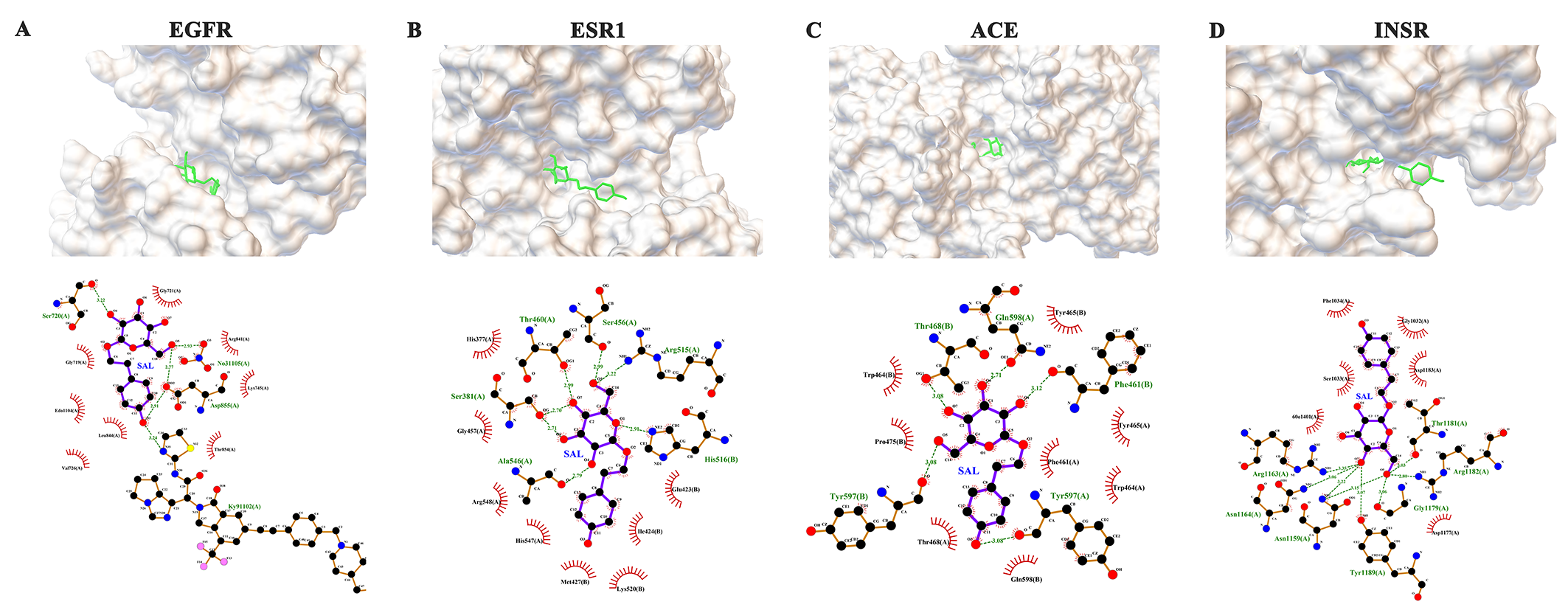

Supplement: Supplementary file 1 [file Image1.TIF]
